# Systematic Investigation of the Efficacy of Sinitang Decoction Against Ulcerative Colitis

**DOI:** 10.3389/fphar.2020.01337

**Published:** 2020-08-31

**Authors:** Enhui Ji, Tingting Wang, Jing Xu, Jianwei Fan, Yi Zhang, Yongxia Guan, Hongjun Yang, Junying Wei, Guimin Zhang, Luqi Huang

**Affiliations:** ^1^School of Traditional Chinese Medicine, Guangdong Pharmaceutical University, Guangzhou, China; ^2^Institute of Chinese Materia Medica, China Academy of Chinese Medical Sciences, Beijing, China; ^3^State Key Laboratory of Generic Manufacture Technology of Chinese Traditional Medicine, Lunan Pharmaceutical Group Co., Ltd., Linyi, China

**Keywords:** ulcerative colitis, Sinitang decoction, C-reactive protein, COL12A1, precise positioning

## Abstract

The aim of this study was to investigate the precise clinical use of Sinitang decoction (SNT) in ulcerative colitis (UC). Network pharmacology-based analysis of the drug components–targets–diseases–pathways was used to predict the possible clinical applications of SNT. Next, 2,4,6-trinitrobenzenesulfonic acid (TNBS) was used to establish a rat model of UC, and the efficacy of SNT against UC was tested, followed by a proteomic analysis of the specific signatures regulated by SNT against UC. SNT was predicted to be effective in inflammatory bowel disease, UC, and several other diseases. In the rats with UC, SNT decreased the disease activity index and colon mucosal damage index compared to the untreated UC model rats. Additionally, SNT reversed the upregulated levels of serum tumor necrosis factor (TNF)-α, prostaglandin E_2_ (PGE_2_), interleukin (IL)-6, and nitric oxide (NO) in UC model rats. The proteomic analysis identified 78 proteins that were differentially regulated by SNT in the rats with UC, which were associated with the Gene Ontology terms sulfur compound binding, calcium ion binding, and Toll-like receptor (TLR)-4 binding. Among these differentially regulated proteins, C-reactive protein (CRP) and collagen alpha-1(XII) chain (COL12A1) were found to be signature proteins associated with the efficacy of SNT against UC. This study represents the first precise investigation of the efficacy and mechanisms of SNT against UC, and shows that SNT is a promising candidate for personalized management of UC.

## Introduction

Ulcerative colitis (UC) is a chronic inflammatory disease of the colon that involves abdominal pain, diarrhea, and hematochezia (Thorsteinsdottir et al., 2011; Eisenstein, 2018). The cause of UC is multifactorial, involving genetic predisposition, epithelial barrier defects, dysregulated immune responses, and environmental factors (Ungaro et al., 2016). The growing incidence of UC has resulted in high hospitalization rates and healthcare costs (Cleynen et al., 2016; Ungaro et al., 2016). In recent years, several drugs, such as aminosalicylates, have been used to ease some of the symptoms of UC, but they have failed to make a remarkable breakthrough to improve the overall clinical picture, and advanced treatments remain urgently needed (Ungaro et al., 2016).

Traditional Chinese medicine, characterized by multiple components and multiple targets, has come a long way to treat many complex diseases. Sinitang decoction (SNT), a common traditional Chinese medicine, consists of three herbs: Fu Zi (*Aconitum carmichaelii Debx*), Gan Jiang (*Rhizoma zingiberis*), and Gan Cao (*Glycyrrhiza uralensis Fisch*) (Liu et al., 2004). SNT has been shown to improve the symptoms of various intestinal diseases, such as inflammatory bowel disease (Pei et al., 2015), diarrhea and irritable bowel syndrome (Chu, 2016; Xi and Wu, 2018), constipation (Zhu, 2016), intestinal mucosal injury (Kexuan et al., 2016), and postoperative intestinal adhesion (Hou, 2004). Additionally, SNT has been shown to reduce the levels of C-reactive protein (CRP), tumor necrosis factor (TNF)-*α*, interleukin (IL)-6, and IL-1 (Zeng et al., 2009; Liu et al., 2014; Cheng et al., 2017). Although some effort has been made to understand the mechanisms of SNT, there remains a need to precisely investigate its efficacy and mechanisms.

Therefore, in this study, an integrative strategy was firstly focused on the clinical positions of SNT against UC. Thereafter, using a rat model of UC to assess the efficacy of SNT, the complex pharmacological mechanisms of SNT were explored. This was followed by conducting a proteomic analysis of the SNT-related differentially regulated proteins that may indicate the efficacy of SNT against UC. This study represents the first precise investigation of the efficacy of SNT against UC, and it shows that SNT is a promising pharmacological approach for the treatment of UC.

## Materials and Methods

### Drugs and Reagents

SNT granules, composed of Fu Zi, Gan Jiang, and Gan Cao based on *Chinese Pharmacopoeia* (2015 edition) in a ratio of 3:2:3, were purchased from the Outpatient Department of Traditional Chinese Medicine of the Chinese Academy of Traditional Chinese Medicine (the chemical structures of the pharmacoactive compounds are shown in the **Figure S1**. Sulfasalazine (SASP) enteric-coated tablets were obtained from Shanghai Xinyi Tianping Pharmaceutical Co., Ltd. (batch no. 09170502; $3.92; Shanghai, China). The 2,4,6-trinitrobenzenesulfonic acid (TNBS) was obtained from Sigma ($397.7; St. Louis, MO, USA). Enzyme-linked immunosorbent assay (ELISA) kits for IL-6, TNF-*α*, prostaglandin E_2_ (PGE_2_), CRP, and collagen alpha-1(XII) chain (COL12A1) were obtained from Cusabio Biotech Co. Ltd. (Wuhan, China), and a nitric oxide (NO) test kit was obtained from Nanjing Jiancheng Bioengineering Institute (Nanjing, China).

### Construction of a Network of Drug Components–Targets–Diseases–Pathways

The names of the three herbs in SNT were submitted to BATMAN-TCM (http://bionet.ncpsb.org/batman-tcm) (Kong et al., 2017; Li et al., 2020). The three herbs were uniformly translated into a list of ingredients (active ingredients) by BATMAN-TCM. The candidate protein targets were then predicted (score cutoff >20). Gene Ontology (GO) functional analysis and Kyoto Encyclopedia of Genes and Genomes (KEGG) pathway analysis were conducted based on the protein targets to determine the GO terms and KEGG pathways that the SNT components may target. The TTD (http://db.idrblab.net/ttd/), OMIM (https://omim.org/), and DAVID 6.8 (https://david.ncifcrf.gov/) databases were used to predict the potential disease targets of SNT.

### Tissue-Specific Distribution

The tissue-specific distribution of the protein targets was determined to explore the potential effect of SNT on the digestive system. The protein targets from BATMAN-TCM were entered into the DAVID 6.8 database. Significant expression sites in the human body (*p*<0.001) were selected for analysis.

### Animal Experiments

Male Sprague–Dawley rats (Beijing Vital River Laboratory Animal Technology Co., Ltd., Beijing, China; license no: SCXK 2016/0006) were used in our experiments. The animal protocol was approved by the Committee on Animal Care and Use of the Institute of Chinese Materia Medica, China Academy of Chinese Medical Sciences. The rats were housed in a room at a temperature of 25 ± 2°C with a light/dark cycle of 12 h/12 h and free access to food and water. All rats were adaptively fed for 7 days before the experiment began. The rats (Jiang et al., 2017; Guo et al., 2020) were then randomly divided into six groups: normal control (n = 8), model (n = 8), high-dose SNT group (19.2 g kg^−1^, n = 6), medium-dose SNT group (9.6 g kg^−1^, equivalent to a normal dose for humans, n = 6), low-dose SNT group (4.8 g kg^−1^, n = 6), and SASP group (0.5 g kg^−1^, n = 6). In all groups except the normal control group, acute UC was induced using one administration of TNBS. Thereafter, rats received 10 mL/kg of each treatment or water daily *via* oral gavage for 10 d. Finally, the rats were anesthetized with 1% pentobarbital sodium, and serum was collected in blank sterile tubes and stored at −80°C. Colon tissue 8 cm from the anus was removed, cut along the mesentery, placed in normal saline to remove the intestinal contents, and then divided equally into two parts, one for hematoxylin and eosin (HE) staining and the other for storage in an Eppendorf tube as a backup were used for the proteomic analysis.

### Measurement of Disease Activity Index (DAI), Colon Mucosal Damage Index (CMDI), and Serum Biochemical Indices

One to three days of TNBS administration and 1–9 days of treatment administration, DAI was comprehensively evaluated by considering the percentage decrease in body mass, stool viscosity, and bleeding status (Keshen et al., 2009; Cotter et al., 2016). Nine days after treatment administration, CMDI was determined based on the colon mucosa morphology, including hyperemia, edema, and erosion (Luk et al., 2002). The levels of IL-6, TNF-*α*, and PGE_2_ in the serum were measured by ELISA according to the manufacturer’s instructions (Cusabio Biotech Co. Ltd.). The level of NO was measured by the Griess method (Weissman and Gross, 1998; Ricart et al., 2002).

### HE Staining of the Colon Tissues

After the colon tissues were fixed in 4% paraformaldehyde for 48 h, they were embedded in paraffin, routinely sectioned, and then subjected to HE staining. Colon tissue injury was observed under a high-magnification microscope (Leica, Wetzlar, Germany).

### Proteomic Analysis

The rat colon tissues were homogenized in 1000 mL phosphate-buffered saline (KCl: 0.2 g, KH_2_PO_4_: 0.2 g, NaCl: 8.0 g, Na_2_HPO_4_•12H_2_O: 3.9054 g, pH 7.4) with a tissue homogenizer. The cells were then lysed to obtain the proteins, and the lysate was centrifuged at 24,000 g for 20 min at 4°C. The supernatant was collected, and the protein concentration was determined by the Bradford method. The proteins were then reduced by dithiothreitol (DTT) for 4 h at 37°C and alkylated by iodoacetamide (IAA) for 60 min at room temperature in the dark. The proteins were then digested using trypsin in 50 mM ammonium bicarbonate (pH 8.0) at a mass ratio of 1:50 enzyme:protein for 24 h at 37°C.

Samples were analyzed on a Q Exactive HF mass spectrometer (Thermo Fisher Scientific, Rockford, IL, USA) connected to an Easy-nLC 1000 liquid chromatography system (Thermo Fisher Scientific). Tryptic peptides were dissolved in a loading buffer (0.1% formic acid and acetonitrile) and eluted with a flow rate of 350 nL/min for 78 min. The MS analysis for QE HF was performed with one full scan (300–1,400 m/z, R=120,000 at 400 m/z) at automatic gain control target of 5×105 ions, followed by up to 30 data-dependent MS/MS scans with higher-energy collision dissociation (target 5×105 ions, max injection time 35 ms, normalized collision energy of 32%), detected in the Orbitrap. The dynamic exclusion of previously acquired precursor ions was enabled at 18 s (Wei et al., 2019).

MaxQuant was used to analyze the proteomics data (Tyanova et al., 2015; Tyanova et al., 2016). Raw MS files was analyzed by MaxQuant software. MS/MS-based peptide identification was carried out with the Andromeda search engine in MaxQuant, Andromeda uses a target–decoy approach to identify peptides and proteins at an FDR <1%. As a forward database, rat protein database from NCBI was used. A reverse database for the decoy search was generated automatically in MaxQuant. Enzyme specificity was set to “Trypsin”, and a minimum number of seven amino acids were required for peptide identification. Oxidation (methionine) and acetylation (protein N-terminal acetylation) were chosen as variable modifications, while cysteine carbamidomethylation was chosen as a fixed modification. Two missed cleavage sites for trypsin were allowed. The iBAQ (intensity-based absolute protein quantification) of each sample were transferred into FOT (a fraction of total protein iBAQ amount per experiment).

Proteomic datasets filtered at different levels for various statistical analyses: Unique peptide number >0, Discard max FOT <0.1 and identified in at least 3 samples. One-way-ANOVA method was used to identify proteins with significant change across different groups (p<0.05 and change ratio≥2).

The interactions among the SNT-related differentially regulated proteins (i.e., proteins that were differentially regulated in the medium-dose SNT group compared to the model group) were investigated using STRING 11.0 (https://string-db.org). A protein–protein interaction network of the differentially regulated proteins was constructed based on the following factors: high reliability, interaction score >0.4, protein–protein interaction enrichment *P*<1.0×10 ^−16^.

### ELISAs

The serum levels of IL-6 (CSB-E04640r), TNF-*α* (CSB-E11987r), PGE_2_ (CSB-E07967r), CRP (CSB-E07922r), and COL12A1 (CSB-EL005719RA) were assessed using ELISA kits. Briefly, the target protein was recognized by the capture antibody, followed by incubation with a horseradish peroxidase-conjugated secondary antibody. Thereafter, colorimetric quantification was conducted be assessing the absorbance at 450 nm using a microplate reader (SpectraMax M5; Molecular Devices, San Jose, CA, USA). The assays were carried out in triplicate for each sample.

## Results

### Prediction of Tissue and Disease Targets of SNT

**Figure 1:**
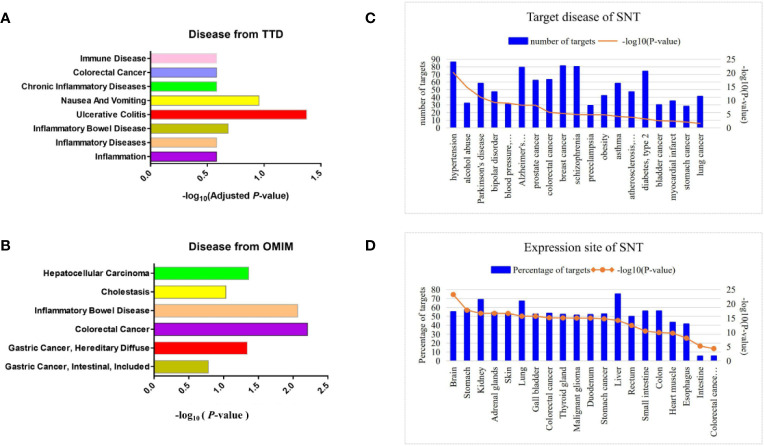
Prediction of disease and tissue targets of SNT. Predicted disease targets of SNT (related to the digestive system) based on the **(A)** TTD, **(B)** OMIM, and **(C)** DAVID analyses. **(D)** Tissue-specific distribution of potential targets of SNT.

Based on BATMAN-TCM, there were 335 chemical components of SNT from Fu Zi (*Aconitum carmichaelii Debx*), Gan Jiang (*Rhizoma zingiberis*), and Gan Cao (*Glycyrrhiza uralensis Fisch*). Next, 1009 corresponding protein targets of SNT were predicted (**Figure S2**). Subsequently, the TTD analysis indicated that SNT was involved in inflammation (18 targets), inflammatory diseases (10 targets), inflammatory bowel disease (6 targets), and UC (5 targets), but only UC was significantly relevant (adjusted *P*<0.05) (**Figure 1A**, **Table S1**), while both the OMIM (**Figure 1B**) and DAVID (**Figure 1C**) analyses showed that SNT may affect colorectal and gastric cancer.

The tissue-specific distribution of the potential protein targets of SNT showed that they were distributed in the liver, kidneys, stomach, colon, and small intestine (**Figure 1D**). Furthermore, the protein targets were enriched in the tissues of digestive system organs and cells, such as colorectal cancer cells, duodenum, gastric cancer cells, rectum and esophagus. The KEGG analysis of the protein targets showed that SNT may affect gastric acid and other secretions, pancreatic secretion, bile secretion, and the association with gastric acid secretion was significant (*P*=4.81×10^−2^) (**Table S2**). In addition, SNT may affect the immunoglobulin A (IgA) intestinal immune network, TLR signaling pathway, NOD-like receptor signaling pathway, and others. In general, the clinical applications of SNT were predicted to involve diseases of the digestive system, particularly UC.

### Assessment of *In Vivo* SNT Efficacy

The *in vivo* pharmacologic effects of SNT against UC *were* evaluated in rats with TNBS-induced UC. Using TNBS, UC was successfully induced, based on the rats’ fur color, defecation, and the presence of hematochezia.

**Figure 2:**
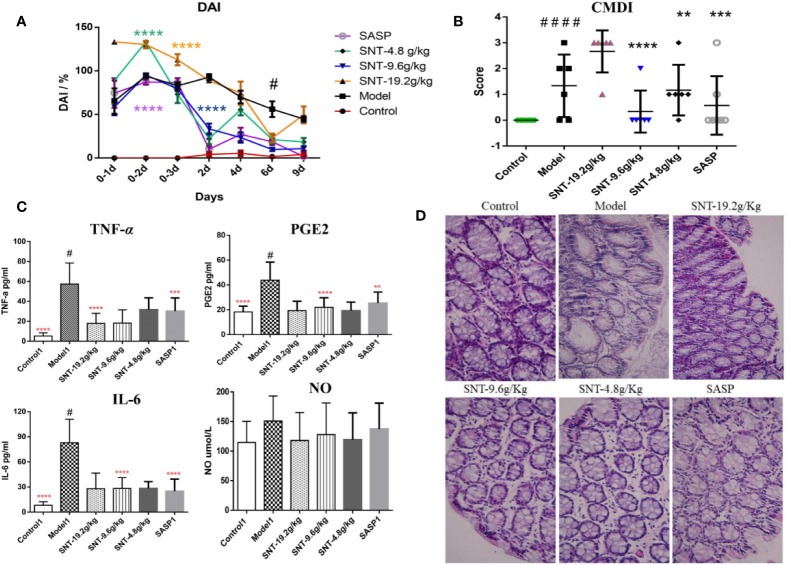
Assessment of the efficacy of SNT against UC in rats with TNBS-induced UC. **(A)** Disease activity index (DAI) in rats in each group (DAI was assessed on the day of the TNBS administration and the next two days (days 0–3, 0–2, and 0–1, where day 0–1 is the first day of TNBS administration) and during treatment administration (days 2, 4, 6, and 9 after treatment began). **(B)** Colon mucosal damage index (CMDI) in rats in each group, evaluated after 9 days of treatment administration. **(C)** Enzyme-linked immunosorbent assays (ELISAs) of serum cytokine levels in rats in each group. **(D)** Hematoxylin and eosin (HE) staining showing the degree of intestinal mucosal injury in rats in each group (×40), **P < 0.01, ***P < 0.001, ****P < 0.0001 vs UC model group; ^#^*P* < 0.05, ^####^*P* < 0.0001 vs normal control group.

As shown in **Figure 2A**, from the second day after TNBS administration, the DAI scores were significantly increased in the modle, SNT, and SASP groups compared to the normal control group. Compared to the model group, both SNT and SASP, except for high-dose SNT, decreased the DAI score as the treatment administration time increased. Among the SNT groups, only medium-dose SNT continued to show decreases in the DAI score from day 1–9 of treatment administration.

Compared to the control group, the modle and low-does SNT group were significantly increased the CMDI score. The CMDI score of the medium-dose SNT group exhibited the same pattern as the SASP group regarding being significantly lower than the CMDI score in the model group and not being significantly different compared to the normal control group. Meanwhile, the CMDI scores only increased for 1 rat in the medium-dose SNT group and 2 rats in the SASP group (**Figure 2B**). However, the DAI and CMDI scores in the high-dose SNT group were similarly as poor (indicating injury) as those in the model group.

Compared to the normal control group, the TNF-*α*, PGE_2_, and IL-6 levels significantly increased in the model group (*P*<0.05) (**Figure 2C**). Compared to the model group, the TNF-α level was decreased in the high-dose SNT group, while the PGE_2_ and IL-6 levels were decreased in the medium-dose SNT and SASP groups. Compared to the SASP group, the TNF-*α*, PGE_2_, and NO levels were decreased in the SNT groups. In addition, although there were no significant differences between groups in the NO level, all the SNT and SASP groups had lower levels than the model group.

The model rats exhibited pathological changes such as mucosal epithelial degeneration, inflammatory cell infiltration, and disordered cell arrangement (with colored brown) compared to the normal control group (**Figure 2D**). While the medium- and low-dose SNT groups exhibited inflammatory cell infiltration with less disordered cell arrangement, the SASP and high-dose SNT groups had inflammatory cell infiltration with more disordered cell arrangement.

Thus, the experimental results in rats indicated that SNT can reduce the levels of TNF-*α*, PGE_2_, and IL-6 in rats with UC and thereby relieve the symptoms of bloody stools, colon mucosal damage, mucosal epithelial degeneration, inflammatory cell infiltration, and disordered cell arrangement (with colored brown).

### Proteomic Analysis of SNT-Related Differentially Regulated Proteins That May Indicate the Efficacy of SNT Against UC

**Figure 3:**
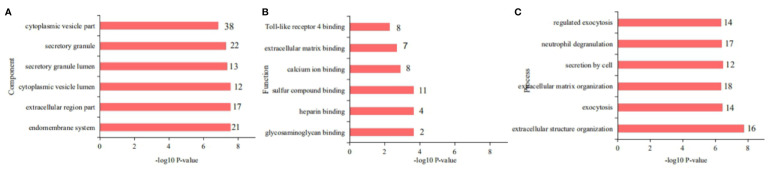
Gene Ontology (GO) classification of the 78 differentially regulated proteins. **(A)** Cellular Components, **(B)** Molecular Functions, and **(C)** Biological Processes.

The SNT-related differentially regulated proteins were investigated by subjecting rat colon tissues to high-throughput proteomic profiling. As a result, 6,110 proteins were identified. After data homogenization and *P* value*<0.05*, 194 proteins were screened and were found to be similarly regulated in the SNT and control groups. Meanwhile, compared to the control group, 78 proteins were differentially expressed in the medium-dose SNT group. Among them, 25 proteins were upregulated and 53 were downregulated in the SNT group (**Table S3**). They were associated with the GO Cellular Component terms cytoplasmic vesicles, secretory granules, extracellular regions, and endomembrane system (**Figure 3A**), the GO Molecular Function terms sulfur compound binding, calcium ion binding, extracellular matrix (ECM) binding, Toll-like receptor (TLR)-4 binding, etc. (**Figure 3B**), and the GO Biological Process terms extracellular structure organization, ECM organization, neutrophil degranulation, etc. (**Figure 3C**). Furthermore, the KEGG pathway analysis showed that SNT may regulate the ECM–receptor interaction, PI3K-Akt signaling pathway, PPAR signaling pathway, and other pathways (**Table S4**), which involved LAMA2, THBS2, HMGCS2, CRP, RXRA VTN, COL12A1, TNN, and other proteins.

**Figure 4:**
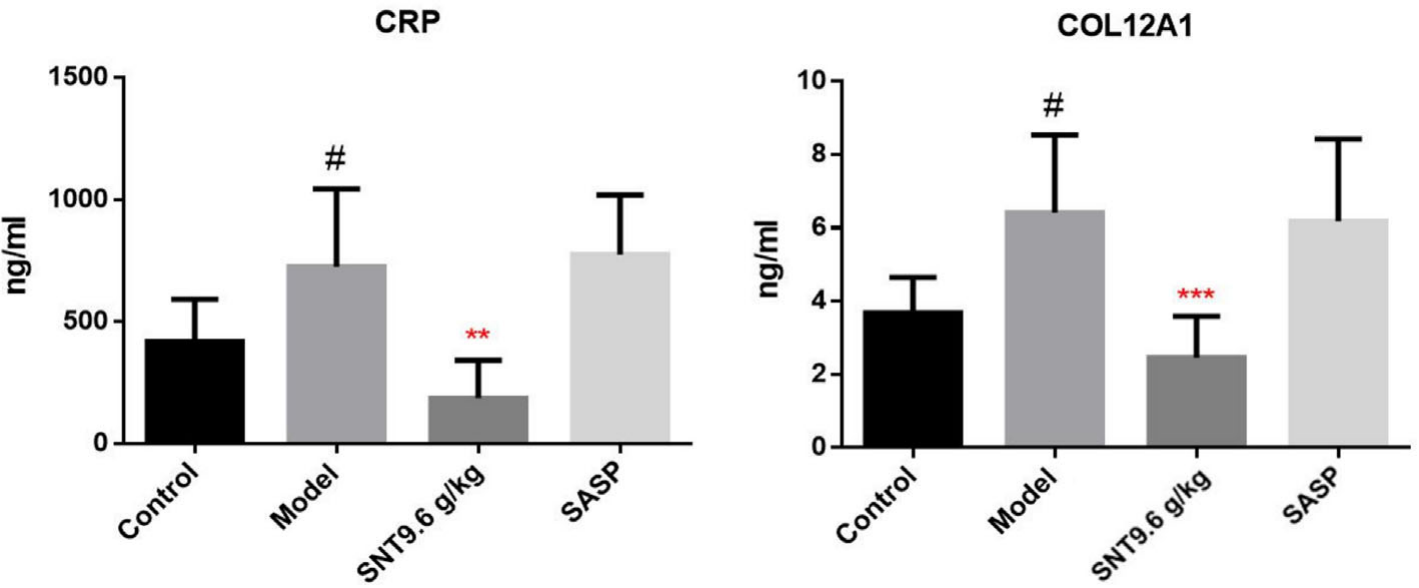
Verification of the SNT-regulated signature proteins (CRP and COL12A1) that may indicate the efficacy of SNT against UC in rats. Serum CRP and COL12A1 levels were quantified by enzyme-linked immunosorbent assays (ELISAs). ^#^*P* < 0.05 vs control group; **P < 0.01 and ***P < 0.001 vs model group.

Candidate signature proteins (CRP and COL12A1) (from among the SNT-related differentially regulated proteins that may indicate the efficacy of SNT against UC) were further verified by ELISA. Compared to the normal control group, the serum CRP and COL12A1 levels increased upon induction of UC by TNBS. As in the proteomic analysis, serum CRP and COL12A1 levels decreased in the medium-dose SNT group compared to the model group, but remained affected by SASP treatment (**Figure 4**). Thus, CRP and COL12A1 are novel signature proteins associated with the efficacy of SNT against UC.

### Interaction Network Involving the Signature Proteins That May Indicate the Efficacy of SNT Against UC

**Figure 5:**
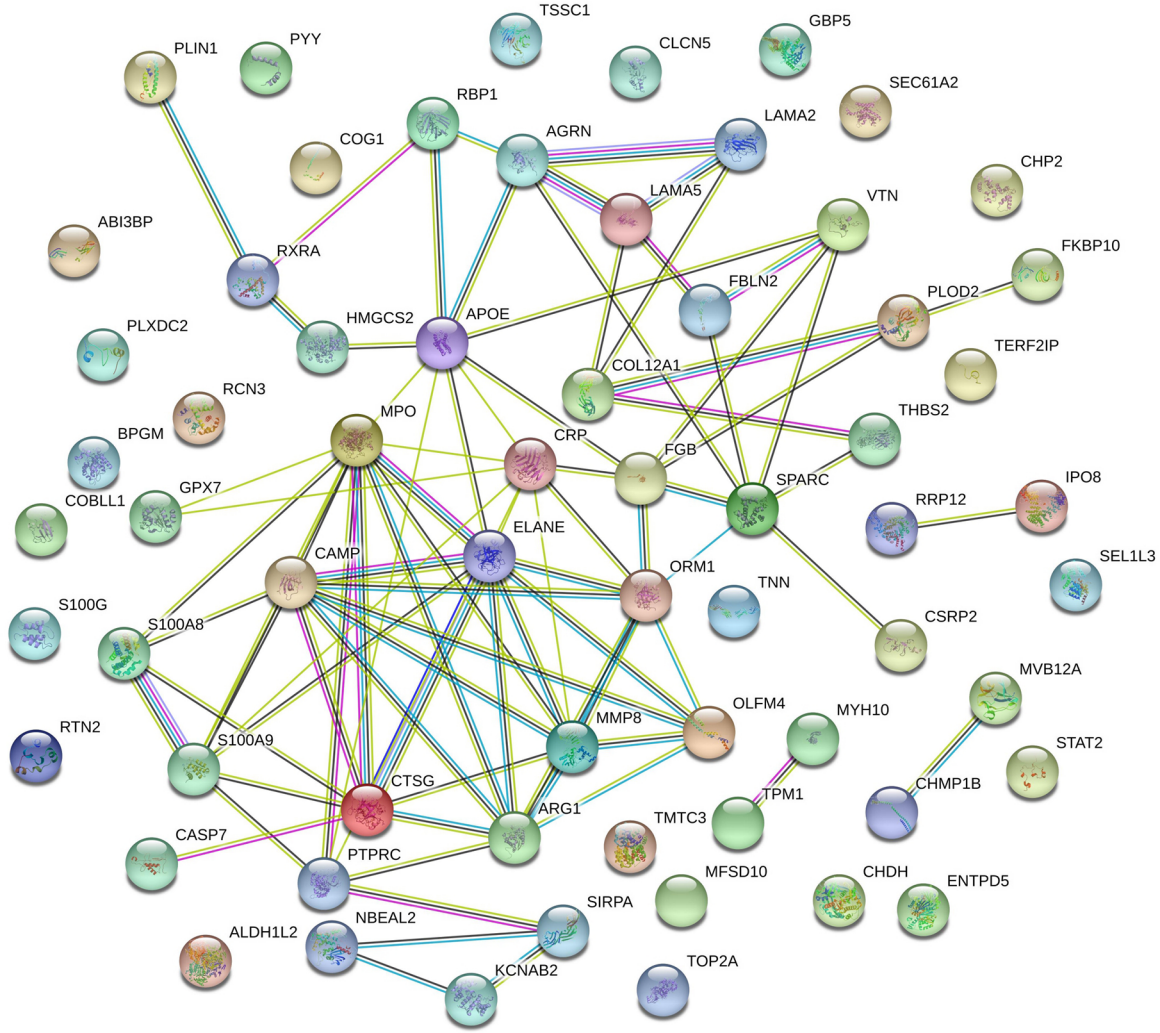
Protein–protein interaction network of SNT-related differentially regulated proteins that may indicate the efficacy of SNT against UC. Each node represents all the proteins produced by a single protein-coding gene locus. Edges represent protein–protein interactions: red line—fusion evidence; green line—neighborhood evidence; blue line—co-occurrence evidence; purple line—experimental evidence; yellow line—text mining evidence; light blue line—database evidence; black line—coexpression evidence.

The interaction network of the 78 differentially regulated proteins was analyzed using STRING. As shown in **Figure 5**, CRP and COL12A1 interact with many proteins, such as ORM1, FGB, PLOD2, THBS2, LAMA5, and LAMA2, and then they have a positive impact on UC.

## Discussion

As a traditional Chinese medicine, although some studies (Hou, 2004; Pei et al., 2015; Chu, 2016; Zhu, 2016) have shown that SNT can act on the digestive system, its precise clinical applications, as well as its pharmacological mechanisms, remain ambiguous. Thus, in this study, these issues were investigated. By utilizing a network pharmacology analysis, not only can we narrow down the candidate target proteins based on experimental data, but we can also use the analysis to predict the network of drug components–targets–diseases–pathways and clearly observe the network of interactions among the candidate signature proteins (Zhang et al., 2019). Furthermore, the proteomic analysis allowed the specific SNT-regulated signature proteins to be investigated.

TNBS is widely used to establish animal models of UC. We observed an obvious physiological injury on the day that TNBS was administered in the rats subjected to TNBS-induced UC, involving loose, bloody stools or stools with occult blood. DAI and CMDI scores are recognized as prominent indicators of acute UC (Luk et al., 2002; Islam et al., 2008; Cotter et al., 2016). In our study, there seemed to be a difference between CMDI and DAI, such as the medium SNT-group in the CMDI was comparable to the controls but the DAI was much worse. Because DAI was assessed on the day of the TNBS administration and the next two days (days -3, -2, and -1, where day 1 is the first day of treatment) and during treatment administration (days 2, 4, 6, and 9 after treatment began), while CMDI was assessed on the day of colon tissue collection (day 9 of treatment administration). Thus, from day 4 of treatment administration in the DAI and CMDI were comparable in the medium-dose SNT and control groups. Moreover, compared to the SASP group, the DAI, CMDI, TNF-α, and PGE_2_ levels in the medium-dose SNT group were nearly the same. However, based on HE staining, the medium-dose and low-dose SNT group had a more obvious improvement than the SASP group regarding inflammatory cell infiltration and cell arrangement.

TNF-*α*, PGE_2_, and IL-6 also play an important role in the inflammatory injury involved in UC (Yamamoto et al., 2000; Gombošová et al., 2011; El Morsy et al., 2015; Sadar et al., 2016; El-Ashmawy et al., 2017; Gawrońska et al., 2017; Rafa et al., 2017; Li et al., 2018; Loynes et al., 2018). Moreover, GO analysis indicated that the proteins regulated by SNT in order to mediate its effects of UC play important roles in the pathogenesis of UC by regulating neutrophil degranulation, cell secretion, and other biological pathways. In fact, neutrophil recruitment is closely related to UC, and it can promote the development of colitis-associated tumorigenesis by activating the IL-1/IL-6 axis (Wang et al., 2014), and participate in the epithelial homeostasis of the colonic epithelial barrier in UC (Sumagin et al., 2013). Overall, SNT depends on its diverse chemical composition and effectively acts on inflammatory factors; this chemical composition reflects an ability to impact various mechanisms/pathways of UC, allowing SNT to exert beneficial effects on UC. In summary, the experimental results based on rats indicated that SNT can improve the symptoms of UC, exerting beneficial pharmacological effects against UC.

Among the SNT-regulated signature proteins that may indicate the efficacy of SNT against UC, CRP is an important parameter of the inflammatory response, and it has been used for guiding treatment and monitoring UC patients (Chang et al., 2015; Zenlea et al., 2016). A previous clinical analysis indicated that CRP levels are associated with colonic tissue inflammation in healthy individuals or patients with mild UC (Rosenberg et al., 2013). In addition, higher CRP levels are significantly associated with requirement for a colectomy, while lower CRP levels are significantly associated with UC remission (Cabriada et al., 2012; Rita et al., 2014). Therefore, CRP can be used to evaluate the severity of UC. Our study also seemed to verify the high expression of CRP in UC, but clinical verification in humans was not conducted.

COL12A1 is a protein that is a member of the fibril-associated collagens with interrupted triple helix (FACIT) collagen family, which is related to the ECM –receptor interaction, PPAR-Peroxisome proliferator-activated receptor and the MAPK-Mitogen-activated protein kinase signaling pathway (Kim et al., 2016; Punetha et al., 2017; Zhang et al., 2018; Xiang et al., 2019). COL12A1 is associated with joint anomalies, gastric cancer, myopathies, ECM defects, and chondromyxoid fibroma. Moreover, in some cases, it is also a specific biomarker of colon and colorectal cancers (Yasuda et al., 2009; Michal et al., 2011; O’Connell et al., 2012; Kalmar et al., 2013; Punetha et al., 2017; Duan et al., 2018; Hauptman et al., 2018; Xiang et al., 2019). Moreover, CRP is already a well-known marker for inflammation in IBD, Our study innovatively discovered the potential effect of COL12A1 on UC. Compared with the CRP, it provided evidence for our study that COL12A1 may be significantly upregulate in UC in rats.

Interestingly, we found that the 1009 predicted protein targets of SNT included CRP but not COL12A1 in BATMAN; COL12A1 may not have been studied very much, as only a few studies have shown its association with Colorectal Cancer (Jingwei et al., 2018; Wu and Xu, 2020) and colon cancer (Dia and Elvira, 2011; Michal et al., 2011). It may be useful as a prognostic marker in the management of UC.

CRP and COL12A1, which were among the 78 SNT-related differentially regulated proteins, did not directly interact but they may interact through their downstream/upstream proteins, such as FBLN2, LAMA5, THBS2 (which may play a crucial role in UC progression and may be a novel prognostic biomarker (Chang et al., 2016; Pekow et al., 2017), and APOE [which may increase susceptibility to UC (Keshen et al., 2009)].

Our study has several limitations. First, the efficacy of SNT was not assessed in human gut-derived cells/tissues (organ culture) to provide an indication of its clinical effects. Second, although the results of this study may inform future research, the precise clinical applications of SNT, as well as its mechanisms, remain unclear.

## Conclusion

In this study, an integrative approach was utilized to address the challenges regarding exploring the clinical applications of SNT. An association between SNT and UC was predicted by a network pharmacology analysis, and the pharmacologic effects of SNT against UC were evaluated in rats with TNBS-induced UC. Also, specific SNT-regulated signature proteins (CRP and COL12A1) were confirmed by the proteomic analysis, which indicated that they may serve as useful signature proteins associated with the efficacy of SNT against UC. In summary, the experimental results based on rats indicated that SNT can improve the symptoms of UC, exerting beneficial pharmacological effects against UC.

## Data Availability Statement

Data are available *via* ProteomeXchange with identifier PXD018509.

## Ethics Statement

The animal study was reviewed and approved by the Committee on Animal Care and Use of the Institute of Chinese Materia Medica, China Academy of Chinese Medical Sciences.

## Author Contributions

EJ, TW, and JX contributed equally to this work. JW, HY, LH, and GZ initiated and designed the project. EJ created the first draft of the manuscript. JW and JX performed major revisions and made comments on the manuscript. JF and YG were responsible for coordinating and supervising the study. TW and YZ performed the animal studies. GZ supported the study. All authors contributed to the article and approved the submitted version.

## Funding

This work was supported by the Key Research and Development Program of Shandong province (2018CXGC1305), the National Science Foundation (81973711) and National Key New Drug Creation and Manufacture Program (2019ZX09201005).

## Conflict of Interest

Author JF, YG and GZ were employed by company Lunan Pharmaceutical Group Co., Ltd.

The remaining authors declare that the research was conducted in the absence of any commercial or financialrelationships that could be construed as a potential conflict of interest.

## Supplementary Material

Supplementary Table 1 Predicted disease targets of SNT (related to the Ulcerative Colitis) based on the TTD.

Supplementary Table 2 Prediction of potential target pathways related to SNT.

Supplementary Table 3 SNT-related differential protein regulation.

Supplementary Table 4 KEGG pathway analysis of differentially regulated proteins related to SNT.

Supplementary Figure 1 The chemical structures of the pharmacoactive compounds of SNT.

Supplementary Figure 2 SNT drug components–targets analysis.

## Abbreviations

UC, Ulcerative colitis; SNT, Sinitang decoction; ELISA, Enzyme-linked immunosorbent assay; KEGG, Kyoto Encyclopedia of Genes and Genomes; HE, Hematoxylin and eosin; LC-MS/MS, Liquid chromatography with tandem mass spectrometry; AGC, automatic gain control; TLR, Toll-like receptor; IgA, Immunoglobulin A; DAI, Disease activity index; CMDI, Colon mucosal damage index; TNF-α, Tumor necrosis factor-α; TNBS, 2,4,6-trinitrobenzenesulfonic acid; IL, Interleukin; PGE_2_, Prostaglandin E_2_; NO, Nitric oxide; DTT, Dithiothreitol; IAA, Iodoacetamide; SASP, Sulfasalazine; GO, Gene Ontology; ECM, Extracellular matrix; CRP, C-reactive protein; COL12A1, Collagen alpha-1(XII) chain; FACIT, Fibril-associated collagens; PPAR, Peroxisome proliferator-activated receptor; MAPK, Mitogen-activated protein kinase.
