# Supplementary material for: Systematic Investigation of the Efficacy of Sinitang Decoction Against Ulcerative Colitis
Source: Front Pharmacol. 2020 Aug 31;11:1337. doi: 10.3389/fphar.2020.01337 (PMC7490561; doi:10.3389/fphar.2020.01337)

**Table S1:Predicted disease targets of SNT (related to the Ulcerative Colitis) based on the TTD.**

| Term description | Adjusted *p*-value | -LOG_10_ | Targets* | ClusterNum | cluster1(p-value\|benjamini-value\|coverage\|EnrichRatio\|gene names) |
| --- | --- | --- | --- | --- | --- |
| Inflammation | 0.262 | 0.581698709 | 18 | 1 | 1.02e-001\|2.62e-001\|18\|1.4\|ADAM8;ADK;ADORA1;ADORA2A;ADRB2;AOC3;CSF2;DHFR;F2R;F2RL1;<br>IKBKB;KIT;MIF;NOS3;PLA2G1B;PPARD;PPARG;PTGS2;\| |
| Inflammatory Diseases | 0.262 | 0.581698709 | 10 | 1 | 1.79e-001\|2.62e-001\|10\|1.4\|ADAM17;ALB;HRH3;IKBKB;IL10;ITGAL;PIK3CD;PTGFR;PTGS2;SYK;<br>\| |
| Inflammatory Bowel Disease | 0.207 | 0.684029655 | 6 | 1 | 4.06e-002\|2.07e-001\|6\|2.2\|ADAM17;ADK;ADORA1;CNR1;P2RX3;PPARG;\| |
| Ulcerative Colitis | 0.0421 | 1.375717904 | 5 | 1 | 3.01e-003\|4.21e-002\|5\|3.9\|HMOX1;MIF;NPR2;PPARG;PTGER4;\| |
| Nausea And Vomiting | 0.111 | 0.954677021 | 4 | 1 | 1.20e-002\|1.11e-001\|4\|3.7\|DRD2;HRH1;HTR3A;HTR6;\| |
| Chronic Inflammatory Diseases | 0.262 | 0.581698709 | 4 | 1 | 6.39e-002\|2.62e-001\|4\|2.4\|MC3R;PPARG;PTGS1;TNF;\| |
| Colorectal Cancer | 0.262 | 0.581698709 | 3 | 1 | 1.19e-001\|2.62e-001\|3\|2.3\|AURKA;FNTA;TLR9;\| |
| Immune Disease | 0.262 | 0.581698709 | 3 | 1 | 1.19e-001\|2.62e-001\|3\|2.3\|MSTN;NT5E;OPRK1;\| |
| Multiple Sclerosis | 0.412 | 0.385102784 | 5 | 1 | 3.34e-001\|4.12e-001\|5\|1.3\|ADRB2;CRH;IFNG;LEP;PPARG;\| |

**Table S2: Prediction of potential target pathways related to SNT**

| KEGG pathway ID | KEGG pathway name | Adjusted p-value | Targets* | gene symbol |
| --- | --- | --- | --- | --- |
| hsa04970 | Salivary Secretion | 0.0923 | 17 | ADRA1A;ADRA1B;ADRA1D;ADRB1;ADRB2;ADRB3;ATP1A1;ATP1A2;ATP2B4;CHRM3; GUCY1B3;ITPR1;ITPR2;ITPR3;KCNMA1;NOS1;RYR3; |
| hsa04971 | Gastric Acid Secretion | 0.0481 | 16 | ATP1A1;ATP1A2;CAMK2D;CAMK2G;CFTR;CHRM3;HRH2;ITPR1;ITPR2;ITPR3; KCNE2;KCNJ1;KCNJ10;KCNJ15;KCNK2;KCNQ1; |
| hsa04972 | Pancreatic Secretion | 0.314 | 15 | AMY2A;ATP1A1;ATP1A2;ATP2A1;ATP2B4;CFTR;CHRM3;ITPR1;ITPR2;ITPR3; KCNMA1;KCNQ1;PLA2G1B;RHOA;RYR2; |
| hsa04973 | Carbohydrate Digestion And Absorption | 0.222 | 9 | AMY2A;ATP1A1;ATP1A2;CACNA1D;GNAT3;MGAM;SI;TAS1R2;TAS1R3; |
| hsa04974 | Protein Digestion And Absorption | 1 | 5 | ATP1A1;ATP1A2;KCNQ1;SLC7A7;SLC8A1; |
| hsa04975 | Fat Digestion And Absorption | 1 | 4 | APOA1;CD36;MTTP;PLA2G1B; |
| hsa04976 | Bile Secretion | 0.0668 | 15 | ABCB1;ABCB4;ABCC4;AQP1;AQP8;ATP1A1;ATP1A2;CFTR;CYP3A4;HMGCR; NR1H4;RXRA;SLC22A1;SLCO1B1;SLCO1B3; |
| hsa04977 | Vitamin Digestion And Absorption | 1 | 2 | APOA1;LRAT; |
| hsa04978 | Mineral Absorption | 0.751 | 7 | ATP1A1;ATP1A2;HMOX1;S100G;SLC26A6;SLC8A1;VDR; |

**Table S4: KEGG pathway analysis of differentially regulated proteins related to SNT.**

| Item name | Item description | Number of differential protein targets | Total number of protein targets | Adjusted *p*-value |
| --- | --- | --- | --- | --- |
| has 04512 | ECM-receptor interaction | 6 | 81 | 0.0000353 |
| has 05146 | Amoebiasis | 4 | 94 | 0.0134 |
| has 04510 | Focal adhesion | 5 | 197 | 0.0155 |
| has 05165 | Human papillomavirus infection | 6 | 317 | 0.0155 |
| has 04151 | PI3K-Akt signaling pathway | 6 | 348 | 0.0187 |
| has 03320 | PPAR signaling pathway | 3 | 72 | 0.0286 |

**Figure S1: The chemical structures of the pharmacoactive compounds of SNT.**


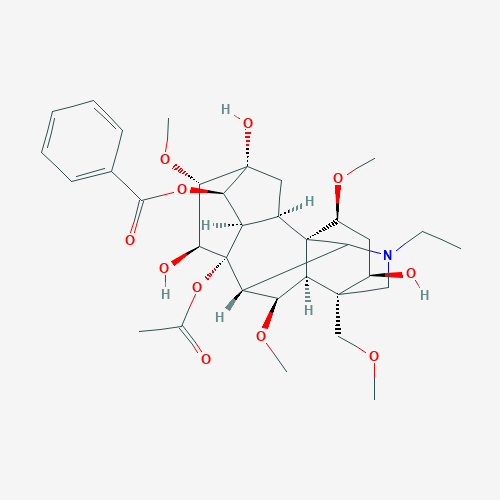

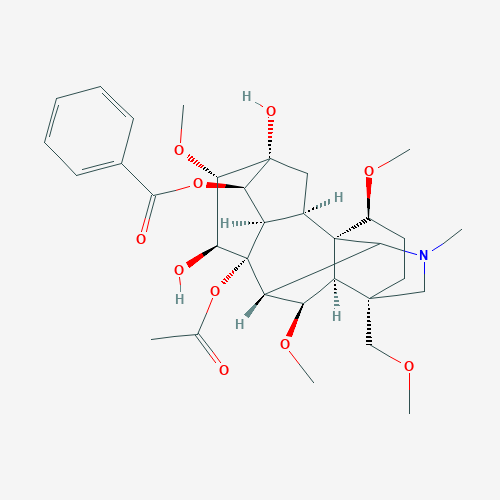

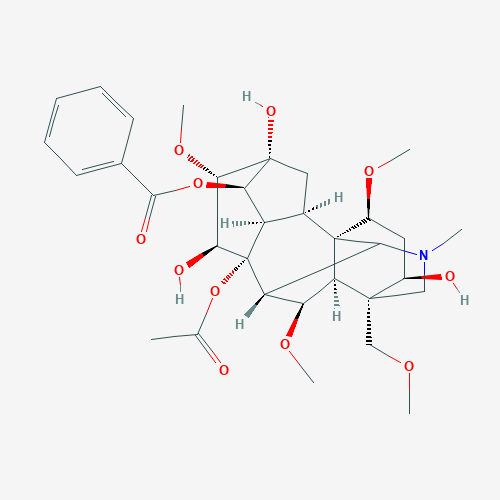

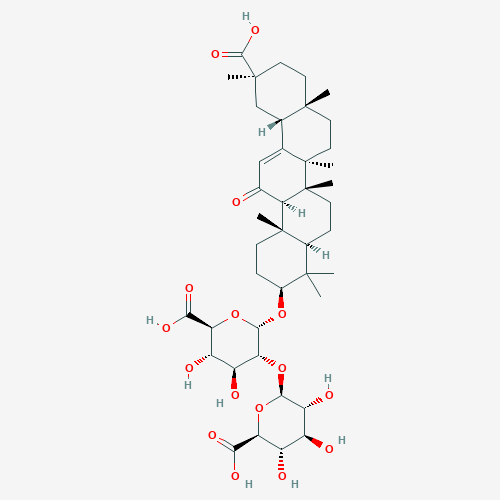

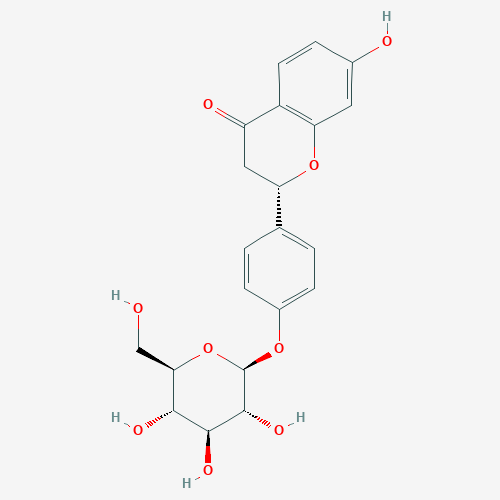


Aconitine Hypaconitine Mesaconitine Glycyrrhizic_acid Liquiritin


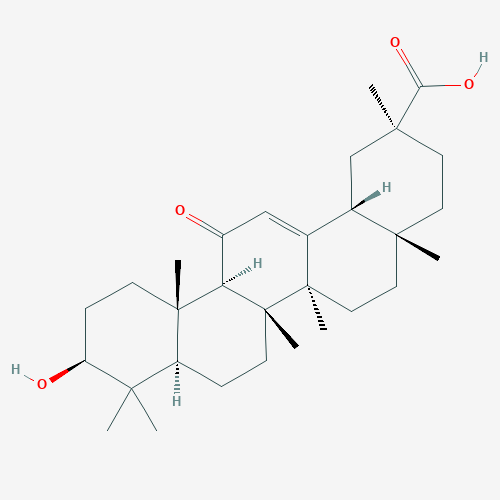

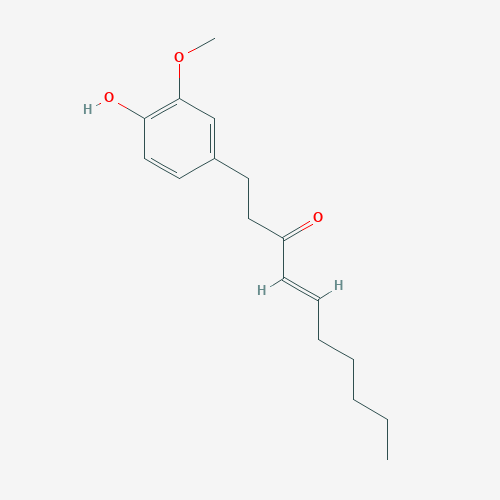

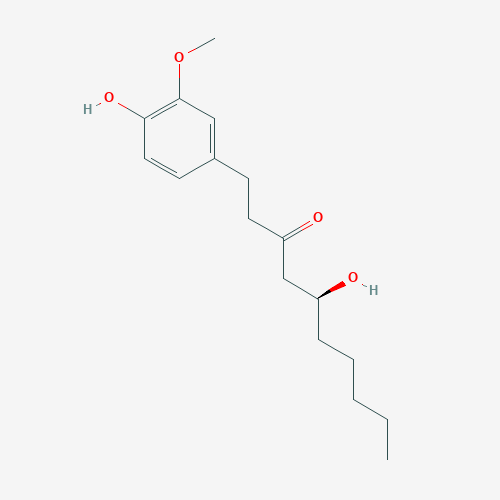


Enoxolone 6-Shogaol 6-Gingerol

**Figure S2: SNT drug components–targets analysis.**


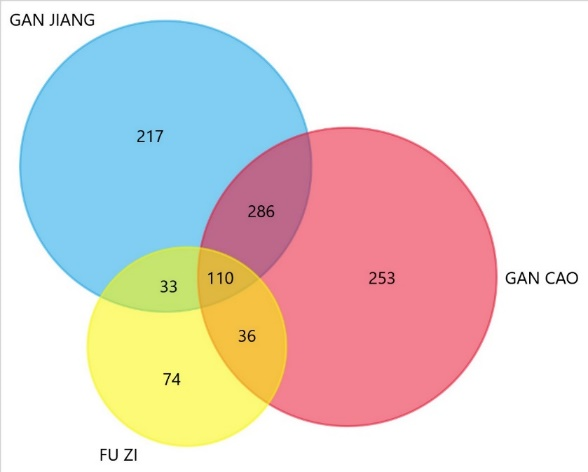

Supplement: Supplementary Table 1 — Predicted disease targets of SNT (related to the Ulcerative Colitis) based on the TTD. [file DataSheet_1.docx]
